# Analyzing the expected values of neighborhood degree-based topological indices in random cyclooctane chains

**DOI:** 10.3389/fchem.2024.1388097

**Published:** 2024-04-26

**Authors:** Liang Jing, Shamaila Yousaf, Saira Farhad, Fairouz Tchier, Adnan Aslam

**Affiliations:** ^1^ General Education Department, Anhui Xinhua University, Hefei, China; ^2^ Department of Mathematics, University of Gujrat, Gujrat, Pakistan; ^3^ Mathematics Department, College of Science, King Saud University, Riyadh, Saudi Arabia; ^4^ Department of Natural Sciences and Humanities, University of Engineering and Technology, Lahore(RCET), Lahore, Pakistan

**Keywords:** chemical graph theory, topological indices, cyclooctane chains, expected values, Randic index

## Abstract

Cyclooctane is classified as a cycloalkane, characterized by the chemical formula *C*_8_ *H*_16_. It consists of a closed ring structure composed of eight carbon atoms and sixteen hydrogen atoms. A cyclooctane chain typically refers to a series of cyclooctane molecules linked together. Cyclooctane and its derivatives find various applications in chemistry, materials science, and industry. Topological indices are numerical values associated with the molecular graph of a chemical compound, predicting certain physical or chemical properties. In this study, we calculated the expected values of degree-based and neighborhood degree-based topological descriptors for random cyclooctane chains. A comparison of these topological indices’ expected values is presented at the end.

## 1 Introduction

Cyclooctane itself is a cyclic molecule, forming a stable ring structure with eight carbon atoms and saturated with hydrogen atoms. One way to modify cyclooctane is by substituting some of its hydrogen atoms with other functional groups, leading to various derivatives with different properties and reactivities. Substituted cyclooctane derivatives can serve as essential building blocks in organic synthesis.

The unique structure and strain of cyclooctane can influence the reactions it undergoes, potentially leading to interesting transformations. Cyclooctane rings can be part of larger molecules, where their strain energy might play a role in the overall reactivity and stability of the molecule. The strain energy in cyclooctane rings, attributed to their angle strain, can make them more reactive in certain reactions, possibly resulting in unexpected products.

Cyclooctane and its derivatives are intriguing subjects for computational chemistry studies, aiding researchers in understanding their structures, energies, and reactivities (Bharadwaj, 2000; Salamci et al., 2006; Alamdari et al., 2008; Ali et al., 2012; Banu et al., 2015). These derivatives find applications in combustion kinetics, drug synthesis, organic synthesis, and more. For instance, cyclooctane-1,2,5,6-tetrol is utilized in the osmium-catalyzed bis-dihydroxylation of 1,5-cyclooctadiene (Salamci et al., 2006). Alamdari (Alamdari et al., 2008), conducted a study on the synthesis of some cyclooctane-based quinoxalines and pyrazines.

The molecular structures, specifically the graphs depicting carbon atoms, in cyclooctanes form cyclooctane systems (also referred to as octagonal systems (Brunvoll et al., 1997)). In these systems, each inner face is enclosed by a regular octagon, and any two octagons are linked by an edge. Let *G* be a graph with a vertex set and an edge set denoted by *V* and *E*. A vertex *v* is called the neighbor of vertex *w* if there is an edge between them (or *vw* ∈ *E*). Let *N*(*v*) denote the set of neighbors of *v*. The degree of vertex *v* is the number of edges incident to it and is denoted by *d*(*v*). We use the notation *δ*(*v*) to denote the neighborhood degree of a vertex *v* and is defined as the sum of the degree of the vertices that are adjacent to *v*, i. e., *δ*(*v*) = *∑*_*u*∈*N*(*v*)_ *d*(*u*). For basic definitions related to graph theory, see (West, 2001).

Topological indices are numerical descriptors that provide information about the connectivity and structure of molecules. Up until now, many topological indices have been proposed by different researchers with applications in chemistry. Among these topological indices, the ones most studied are those based on the degree of vertices in a graph. Milan Randic introduced the first degree-based topological index known as the branching index (Randic, 1975). Randic noted that this index is well-suited for assessing the degree of branching within the carbon atom skelton of saturated hydrocarbons. For a graph *G*, the Randic index is the sum of $\frac{1}{\sqrt{d_{v}d_{w}}}$ over all edges *vw* ∈ *E*, i.e.,

$$R\left(G\right)=\Sigma_{vw\in E}\frac{1}{\sqrt{d_{v}d_{w}}}.$$

The Randic index shows strong correlations with various physico-chemical properties of alkanes, including but not limited to boiling points, enthalpies of formation, chromatographic retention periods, surface areas, and parameters in the Antoine equation for vapor pressure (Kier et al., 1975).

The second Gourava index was proposed by Kulli (Kulli, 2017), in 2017 and is defined as

$$GO_{2}\left(G\right)=\Sigma_{vw\in E}\left(d_{v}+d_{w}\right)d_{v}d_{w}.$$

Recently, Mondal et al. (Das and Trinajstic, 2010; Imran et al., 2017; Ali et al., 2019), proposed some topological indices based on neighborhood degree. The modified neighborhood forgotten index of a graph *G* is denoted by $F_{G}^{*}$ and has the mathematical formula

$$F_{N}^{*}\left(G\right)=\underset{uv\in E\left(G\right)}{\sum }\delta {\left(u\right)}^{2}+\delta {\left(v\right)}^{2}.$$ (1)

The second modified neighborhood Zagreb index of a graph *G* is defined as

M2nmG=∑uv∈EG1δuδv. (2)

It was observed that these two topological descriptors show a very good correlation with two physical properties, namely, the acentric factor and the entropy of the octane isomers. Therefore, these topological descriptors are of chemical importance. In, Mondal et al. proposed a few more topological descriptors based on neighborhood degree. He named these topological descriptors the third NDe index and the fourth NDe index. These topological indices are defined as

$$ND_{3}\left(G\right)=\underset{uv\in E\left(G\right)}{\sum }\delta \left(u\right)\delta \left(v\right)\left(\delta \left(u\right)+\delta \left(v\right)\right),$$ (3)

$$ND_{4}\left(G\right)=\underset{uv\in E\left(G\right)}{\sum }{\left(\delta \left(u\right)\delta \left(v\right)\right)}^{-\frac{1}{2}}.$$ (4)

Different researchers have studied the expected values of random molecular structures in the recent past. Raza et al. (Raza et al., 2023a), conducted calculations for the expected values of sum-connectivity, harmonic, Sombor, and Zagreb indices in cyclooctane chains. In the work presented in (Raza, 2022), expected values for the harmonic and second Zagreb indices were determined for random spiro chains and polyphenyl. Additionally, Raza et al. (Raza et al., 2023b), computed the expected value of the first Zagreb connection index in random cyclooctane chains, random polyphenyl chains, and random chain networks. Explicit formulas for the expected values of certain degree-based topological descriptors of random phenylene chains were provided by Hui et al. (Hui et al., 2023). Zhang et al. (Zhang et al., 2018), discussed the topological indices of generalized bridge molecular graphs, while in separate works (Zhang et al., 2022; Zhang et al., 2023), they computed the topological indices of some supramolecular chains using graph invariants. For more details on this topic of research, readers can see the following papers (Mondal et al., 2019; Xu et al., 2020; Mondal et al., 2021; Raza, 2021).

The main aim of this work is to find the expected values of the Randic index, the second Gourava index, the modified neighborhood forgotten index, the third degree neighborhood index, and the fourth degree neighborhood index of the random cyclooctane chain. Moreover, we give a comparison between the expected values of these topological indices.

## 2 Expected values of topological descriptors for random cyclooctane chains

**FIGURE 1:**
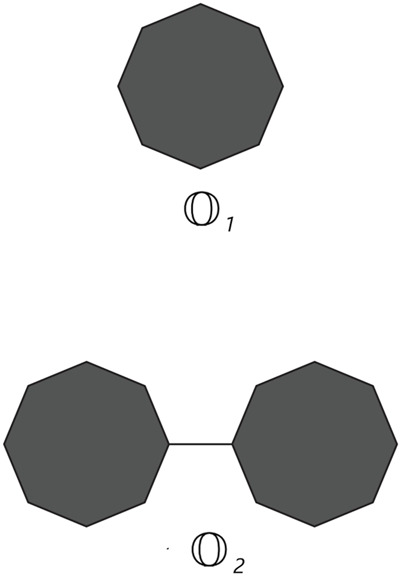
cyclooctane chains with single and double octagons.

**FIGURE 2:**
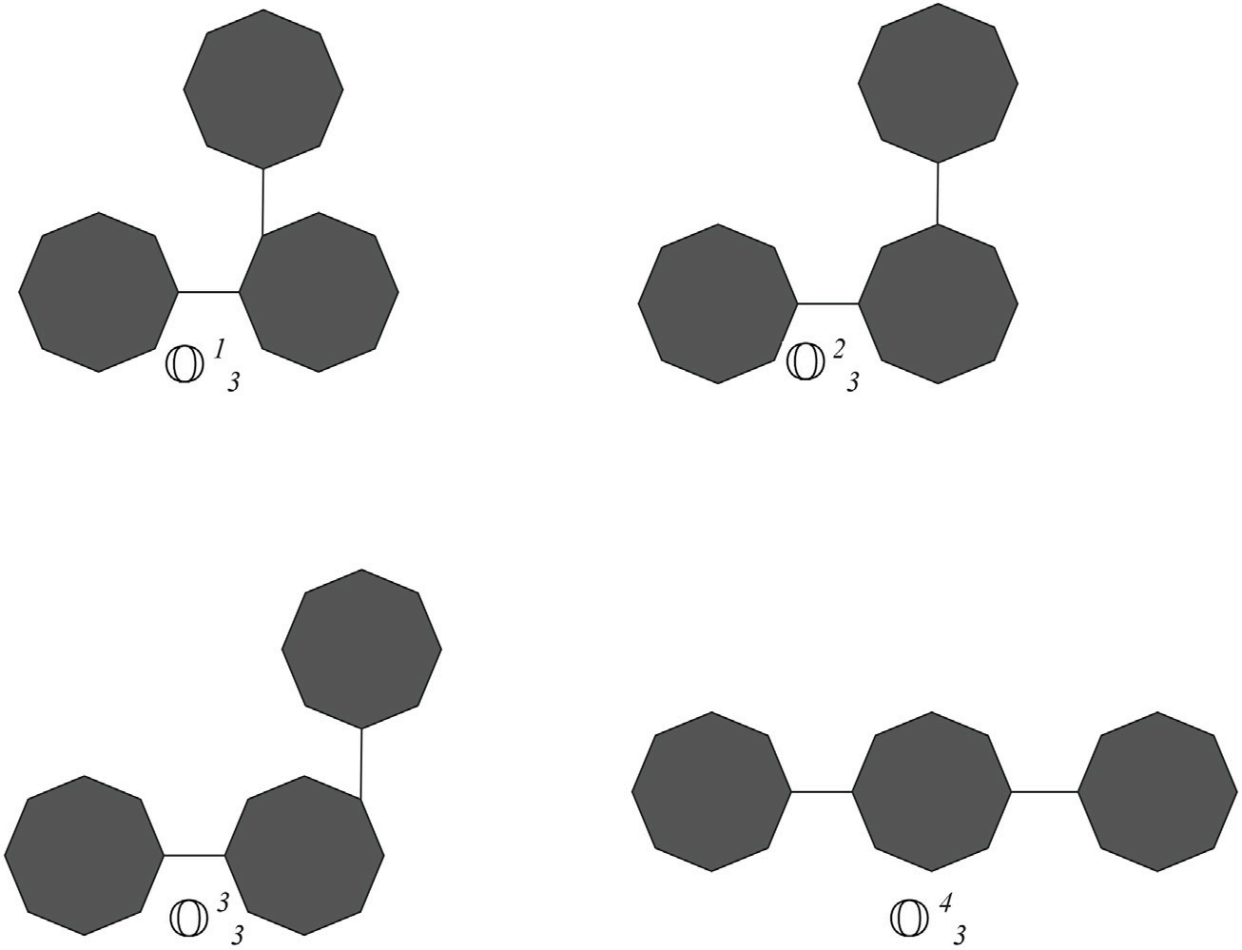
cyclooctane chains with *t* =3.

**FIGURE 3:**
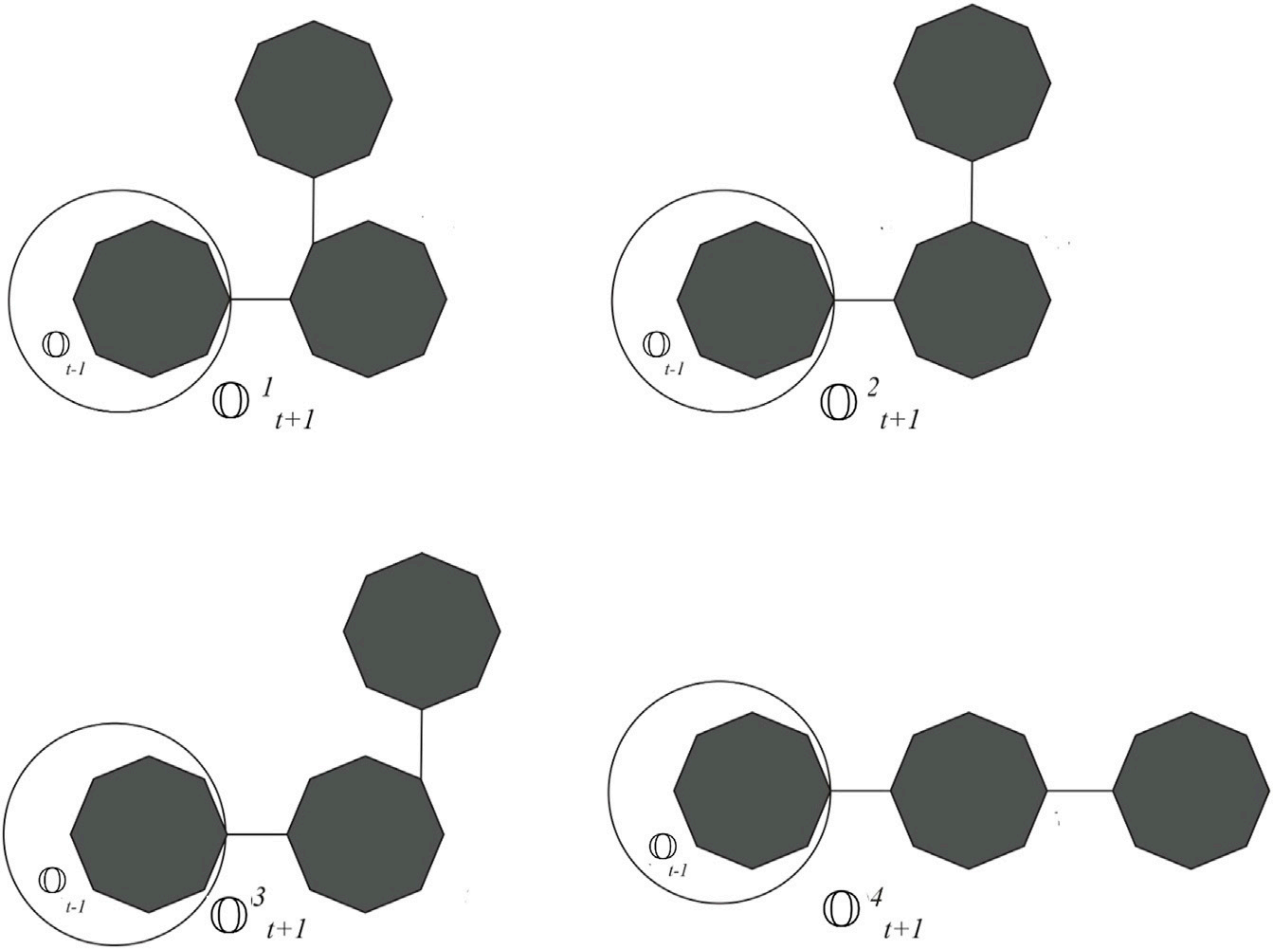
cyclooctane chains with *t* >3.

Cyclooctane is a cyclic hydrocarbon with eight carbon atoms arranged in a ring. While it does not form chains itself, neighboring cyclooctane molecules can interact through intermolecular forces. Understanding these interactions is crucial for studying the physical properties and behavior of cyclooctane and similar cycloalkanes. Cyclooctane graphs are examples of cyclic graphs, which are graphs containing a single cycle as their main structural component. A random cyclooctane chain with a length of *t* is obtained by connecting *t* octagons in a linear arrangement, where any two consecutive octagons are randomly joined by an edge between vertices. We use the notation $O_{t}$ to represent a random cyclooctane chain containing *t* octagons (of length *t*). Observe that there is a unique cyclooctane chain for *t* = 1, 2 (see Figure 1). For *t* ≥ 3, at each step, two octagons can be attached to each other by an edge in four different ways, which results in a random cyclooctane chain $O_{t}$ (see Figure 2). Suppose *p*_1_, *p*_2_, *p*_3_, and *p*_4_ are the probabilities of attaching the octagons at these four places. We call the corresponding cyclooctane chain with probability *p*_*i*_ as $O_{t}^{p_{i}},1\leq i\leq 4$ (see Figure 3). The four possible constructions at each step are as follows:

(a) $O_{t-1}\to O_{t}^{p_{1}}$ with probability *p*_1_,
(b) $O_{t-1}\to O_{t}^{p_{2}}$ with probability *p*_2_,
(c) $O_{t-1}\to O_{t}^{p_{3}}$ with probability *p*_3_,
(d) $O_{t-1}\to O_{t}^{p_{4}}$ with probability *p*_4_ = (1 − *p*_1_ − *p*_2_ − *p*_3_), with probability.

From the graph of the cyclooctane chain, it is easy to see that there are only (2,2), (2,3), and (3,3) types of edges. Let *x*_*ij*_ denote the number of edges of $O_{t}$ with end vertices of degrees *i* and *j*, respectively. By using the definition, the expressions for the Randic index and the second Gourava index are as follows:

$$R\left(O_{t}\right)=\frac{1}{2}x_{22}+\frac{1}{\sqrt{6}}x_{23}+\frac{1}{3}x_{33}.$$ (5)

$$GO_{2}\left(O_{t}\right)=16x_{22}+30x_{23}+54x_{33}.$$ (6)

Since $O_{t}$ is a random cyclooctane chain, it follows that $R\left(O_{t}\right)$ and $GO_{2}\left(O_{t}\right)$ are random variables. We use the notations $E^{R}\left(O_{t}\right)$ and $E^{GO_{2}}\left(O_{t}\right)$ to denote the expected values of the random cyclooctane chain $O_{t}$ . In the next theorem, we give an explicit expression for the expected value of the Randic index for the cyclooctane chain $O_{t}$ .

Theorem 2.1. Let $O_{t}$ be a random cyclooctane chain of length *t* ≥ 2. Then,

$$E^{R}\left(O_{t}\right)=p_{1}\left(\frac{15-2\sqrt{6}}{6}t-\frac{15-2\sqrt{6}}{3}\right)+\left(\frac{7+2\sqrt{6}}{3}\right)t+\frac{15-2\sqrt{6}}{3}.$$

Proof. Let *t* = 2, then $E\left(O_{2}\right)=\frac{19+2\sqrt{6}}{3}$ , which is indeed true. For *t* ≥ 3, there are four possibilities.

A) If $O_{t-1}\to O_{t}^{p_{1}}$ with probability *p*_1_, then $x_{22}\left(O_{t}^{p_{1}}\right)=x_{22}\left(O_{t-1}\right)+5$ , $x_{23}\left(O_{t}^{p_{1}}\right)=x_{23}\left(O_{t-1}\right)+2$ and $x_{33}\left(O_{t}^{p_{1}}\right)=x_{33}\left(O_{t-1}\right)+2$ . Substituting these values into equation 5 yields $R\left(O_{t}^{p_{1}}\right)=R\left(O_{t-1}\right)+\frac{19+2\sqrt{6}}{6}$ .
B) If $O_{t-1}\to O_{t}^{p_{2}}$ with probability *p*_2_, then $x_{22}\left(O_{t}^{p_{2}}\right)=x_{22}\left(O_{t-1}\right)+4$ , $x_{23}\left(O_{t}^{p_{2}}\right)=x_{23}\left(O_{t-1}\right)+4$ and $x_{33}\left(O_{t}^{p_{2}}\right)=x_{33}\left(O_{t-1}\right)+1$ . Substituting these values into equation 5 yields $R\left(O_{t}^{p_{2}}\right)=R\left(O_{t-1}\right)+\frac{7+2\sqrt{6}}{3}$ .
C) If $O_{t-1}\to O_{t}^{p_{3}}$ with probability *p*_3_, then $x_{22}\left(O_{t}^{p_{2}}\right)=x_{22}\left(O_{t-1}\right)+4$ , $x_{23}\left(O_{t}^{p_{2}}\right)=x_{23}\left(O_{t-1}\right)+4$ and $x_{33}\left(O_{t}^{p_{2}}\right)=x_{33}\left(O_{t-1}\right)+1$ . Substituting these values into equation 5 yields $R\left(O_{t}^{p_{3}}\right)=R\left(O_{t-1}\right)+\frac{7+2\sqrt{6}}{3}$ .
D) If $O_{t-1}\to O_{t}^{p_{4}}$ with probability *p*_4_ = (1 − *p*_1_ − *p*_2_ − *p*_3_), then $x_{22}\left(O_{t}^{p_{4}}\right)=x_{22}\left(O_{t-1}\right)+4$ , $x_{23}\left(O_{t}^{p_{3}}\right)=x_{23}\left(O_{t-1}\right)+4$ and $x_{33}\left(O_{t}^{p_{3}}\right)=x_{33}\left(O_{t-1}\right)+1$ . Substituting these values into equation 5 yields $R\left(O_{t}^{p_{4}}\right)=R\left(O_{t-1}\right)+\frac{7+2\sqrt{6}}{3}$ .

Now, we have

$$\begin{array}{ccc} E^{R}\left(O_{t}\right) & = & p_{1}R\left(O_{t}^{p_{1}}\right)+p_{2}R\left(O_{t}^{p_{2}}\right)+p_{3}R\left(O_{t}^{p_{3}}\right)+p_{4}R\left(O_{t}^{p_{4}}\right)\\ & = & p_{1}\left(R\left(O_{t-1}^{p}\right)+\frac{19+2\sqrt{6}}{6}\right)+p_{2}\left(R\left(O_{t-1}^{p}\right)+\frac{7+2\sqrt{6}}{3}\right)\\ & & +p_{3}\left(R\left(O_{t-1}^{p}\right)+\frac{7+2\sqrt{6}}{3}\right)+p_{4}\left(R\left(O_{t-1}^{p}\right)+7+2\sqrt{6}3\right). \end{array}$$

By employing the operator *E* on both sides and considering the fact that $E\left[E^{R}\left(O_{t}\right)\right]=E^{R}\left(O_{t}\right)$ , we get

$$ER\left(O_{t}\right)=ER\left(O_{t-1}\right)+\frac{19+2\sqrt{6}}{6}p_{1}+\frac{7+2\sqrt{6}}{3}p_{2}+\frac{7+2\sqrt{6}}{3}p_{3}+\frac{7+2\sqrt{6}}{3}p_{4}.$$ (7)

Finally, solving the recurrence relation (7), we obtain

$$E^{R}\left(O_{t}\right)=p_{1}\left(\frac{15-2\sqrt{6}}{6}t-\frac{15-2\sqrt{6}}{3}\right)+\frac{7+2\sqrt{6}}{3}+\frac{15-2\sqrt{6}}{3}.$$

Theorem 2.2. Let $O_{t}$ be a random cyclooctane chain of length *t* ≥ 2. Then

$$E^{GO_{2}}\left(O_{t}\right)=10p_{1}\left(t-2\right)+238t-110.$$

Proof. Let *t* = 2, then *E*_2_ = 366, which is indeed true. For *t* ≥ 3, there are four possibilities

A) If $O_{t-1}\to O_{t}^{p_{1}}$ with probability *p*_1_, then $x_{22}\left(O_{t}^{p_{1}}\right)=x_{22}\left(O_{t-1}\right)+5$ , $x_{23}\left(O_{t}^{p_{1}}\right)=x_{23}\left(O_{t-1}\right)+2$ and $x_{33}\left(O_{t}^{p_{1}}\right)=x_{33}\left(O_{t-1}\right)+2$ . Using these values in Eq. 6 we have $GO_{2}\left(O_{t}^{p_{1}}\right)=GO_{2}\left(O_{t-1}\right)+248$ .
B) If $O_{t-1}\to O_{t}^{p_{2}}$ with probability *p*_2_, then $x_{22}\left(O_{t}^{p_{2}}\right)=x_{22}\left(O_{t-1}\right)+4$ , $x_{23}\left(O_{t}^{p_{2}}\right)=x_{23}\left(O_{t-1}\right)+4$ and $x_{33}\left(O_{t}^{p_{2}}\right)=x_{33}\left(O_{t-1}\right)+1$ . Using these values in Eq. 6 we have $GO_{2}\left(O_{t}^{p_{2}}\right)=GO_{2}\left(O_{t-1}\right)+238$ .
C) If $O_{p}^{t-1}\to O_{t}^{p_{3}}$ with probability *p*_3_, then $x_{22}\left(O_{t}^{p_{2}}\right)=x_{22}\left(O_{t-1}\right)+4$ , $x_{23}\left(O_{t}^{p_{2}}\right)=x_{23}\left(O_{t-1}\right)+4$ and $x_{33}\left(O_{t}^{p_{2}}\right)=x_{33}O_{t-1}+1$ . Using these values in Eq. 6 we have $GO_{2}\left(O_{t}^{p_{3}}\right)=GO_{2}\left(O_{t-1}\right)+238$ .
D) If $O^{t-1}\to O_{t}^{p_{4}}$ with probability *p*_4_ = (1 − *p*_1_ − *p*_2_ − *p*_3_), then $x_{22}\left(O_{t}^{p_{4}}\right)=x_{22}\left(O_{t-1}\right)+4$ , $x_{23}\left(O_{t}^{p_{3}}\right)=x_{23}\left(O_{t-1}\right)+4$ and $x_{33}\left(O_{t}^{p_{3}}\right)=x_{33}\left(O_{t-1}\right)+1$ . Using these values in Eq. 6 we have $GO_{2}\left(O_{t}^{p_{4}}\right)=GO_{2}\left(O_{t-1}^{p}\right)+238$ .

Thus, we obtain

$$\begin{array}{ccc} GO_{2}\left(O_{t}^{p}\right) & = & p_{1}GO_{2}\left(O_{t}^{p_{1}}\right)+p_{2}GO_{2}\left(O_{t}^{p_{2}}\right)+p_{3}GO_{2}\left(O_{t}^{p_{3}}\right)+p_{4}GO_{2}\left(O_{t}^{p_{4}}\right)\\ & = & p_{1}\left(GO_{2}\left(O_{t-1}\right)+248\right)+p_{2}\left(GO_{2}\left(O_{t-1}\right)+238\right)+p_{3}\left(GO_{2}\left(O_{t-1}\right)+238\right)\\ & + & p_{4}\left(GO_{2}\left(O_{t-1}^{p}\right)+238\right).\\ GO_{2}\left(O_{t}^{p}\right) & = & GO_{2}\left(O_{t-1}^{p}\right)+248p_{1}+238p_{2}+238p_{3}+238p_{4}. \end{array}$$

By employing the operator *E* on both sides and considering the fact that $E\left[E^{GO_{2}}\left(O_{t}\right)\right]=E^{GO_{2}}\left(O_{t}\right)$ , we get

$$E^{GO_{2}}\left(O_{t}\right)=E^{GO_{2}}\left(O_{t-1}\right)+238+10p_{1}.$$ (8)

Finally, solving the recurrence relation (8), we obtain

$$E^{GO_{2}}\left(O_{t}\right)=10p_{1}\left(t-2\right)+238t-110.$$

**FIGURE 4:**
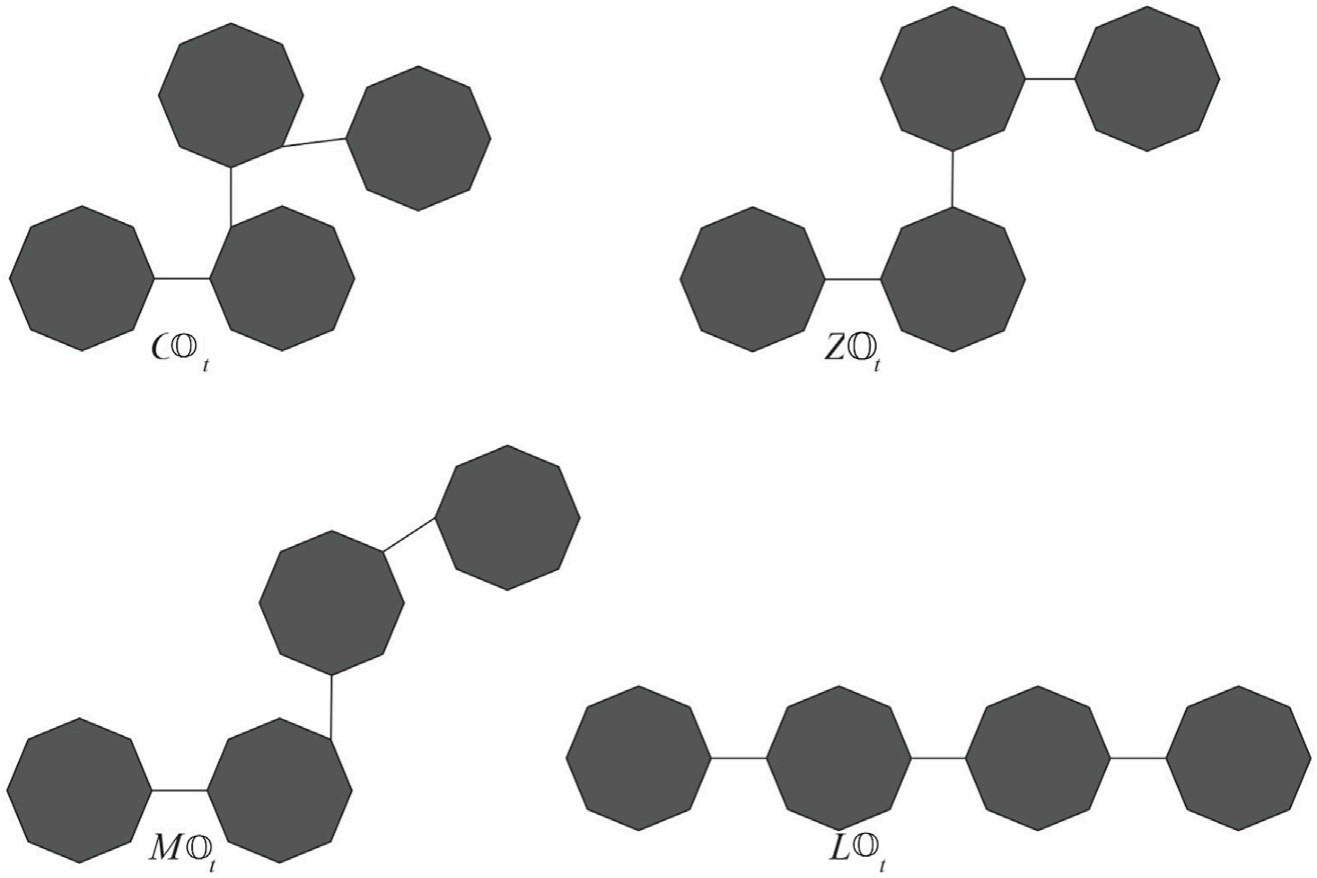
some special cases of cyclooctane chains $CO_{t},ZO_{t},MO_{t},LO_{t}$ .

If the probability is invariable to the step parameter and constant, then this process is called a zeroth-order Markov process. We obtain some special classes of cyclooctane chains if we take one of the values of *p*_1_,*p*_2_, *p*_3_, and *p*_4_ as one. Let $CO_{t}$ , $ZO_{t}$ , $MO_{t}$ , and $LO_{t}$ (see Figure 4) be the classes of cyclooctane chains obtained by taking *p*_1_ = 1, *p*_2_ = 1, *p*_3_ = 1, and *p*_4_ = 1, respectively. The following corollary is an immediate consequence of Theorems 2.1 and 2.2.

Corollary 2.3. *Let* *t* ≥ 2*, then*

1.
• $E^{R}\left(CO_{t}\right)=\left[\frac{19+2\sqrt{6}}{6}\right]t$ .
  • ${\;E}^{R}\left(ZO_{t}\right)=\left[\frac{7+2\sqrt{6}}{3}\right]t+\frac{15-2\sqrt{6}}{3}$ .
  • ${\;E}^{R}\left(MO_{t}\right)=\left[\frac{7+2\sqrt{6}}{3}\right]t+\frac{15-2\sqrt{6}}{3}$ .
  • ${\;E}^{R}\left(LO_{t}\right)=\left[\frac{7+2\sqrt{6}}{3}\right]t+\frac{15-2\sqrt{6}}{3}$ .
2.
• $E^{GO_{2}}\left(CO_{t}\right)=248t-130$ .
  • ${\;E}^{GO_{2}}\left(ZO_{t}\right)=238t-110$ .
  • ${\;E}^{GO_{2}}\left(MO_{t}\right)=238t-110$ .
  • ${\;E}^{GO_{2}}\left(LO_{t}\right)=238t-110$ .

Next, we compute the expected values of topological indices depending on neighborhood degree. For this, we need to find the partition of the edge set of $O_{t}$ based on the neighborhood degree of the end vertices of each edge. Observe that there are only (4,4), (4,5), (5,5), (5,7), (5,8), (6,7), (7,7), (7,8), and (8,8) types of edges based on neighborhood degree in $O_{t}$ . We use the notation *y*_*ij*_ to denote the number of edges of $O_{t}$ whose end vertices have neighborhood degrees *i* and *j*, respectively. For *t* = 3, it is easy to calculate that $y_{44}\left(O_{t}^{p_{1}}\right)=11$ , $y_{45}\left(O_{t}^{p_{1}}\right)=6$ , $y_{57}\left(O_{t}^{p_{1}}\right)=4\;y_{58}\left(O_{t}^{p_{1}}\right)=2$ , $y_{78}\left(O_{t}^{p_{1}}\right)=2$ , $y_{88}\left(O_{t}^{p_{1}}\right)=1$ , $y_{44}\left(O_{t}^{p_{2}}\right)=10$ , $y_{45}\left(O_{t}^{p_{2}}\right)=6$ , $y_{57}\left(O_{t}^{p_{2}}\right)=6$ , $y_{67}\left(O_{t}^{p_{2}}\right)=2$ , $y_{77}\left(O_{t}^{p_{2}}\right)=1$ , $y_{44}\left(O_{t}^{p_{3}}\right)=9$ , $y_{45}\left(O_{t}^{p_{3}}\right)=6$ , $y_{55}\left(O_{t}^{p_{3}}\right)=1$ , $y_{57}\left(O_{t}^{p_{3}}\right)=8$ , $y_{77}\left(O_{t}^{p_{3}}\right)=2\;y_{44}\left(O_{t}^{p_{4}}\right)=8$ , $y_{45}\left(O_{t}^{p_{4}}\right)=8$ , $y_{57}\left(O_{t}^{p_{4}}\right)=8$ , and $y_{77}\left(O_{t}^{p_{4}}\right)=2$ . The expressions for the neighborhood degree based topological indices are as follows

$$\begin{array}{ll} F_{N}^{*}\left(O_{t}\right) & =32y_{44}\left(O_{t}\right)+41y_{45}\left(O_{t}\right)+50y_{55}\left(O_{t}\right)+74y_{57}\left(O_{t}\right)+89y_{58}\left(O_{t}\right)+85y_{67}\left(O_{t}\right)\\ & \;+98y_{77}\left(O_{t}\right)+113y_{78}\left(O_{t}\right)+128y_{88}\left(O_{t}\right). \end{array}$$ (9)

M2nmOt=116y44Ot+120y45Ot+125y55Ot+135y57Ot+140y58Ot+142y67Ot+149y77Ot+156y78Ot+164y88Ot. (10)

$$\begin{array}{ll} ND_{3}\left(O_{t}\right) & =128y_{44}\left(O_{t}\right)+180y_{45}\left(O_{t}\right)+250y_{55}\left(O_{t}\right)+420y_{57}\left(O_{t}\right)+520y_{58}\left(O_{t}\right)+546y_{67}\left(O_{t}\right)\\ & \;+686y_{77}\left(O_{t}\right)+840y_{78}\left(O_{t}\right)+1024y_{88}\left(O_{t}\right). \end{array}$$ (11)

$$\begin{array}{ll} ND_{4}\left(O_{t}\right) & =\frac{1}{4}y_{44}\left(O_{t}\right)+\frac{1}{2\sqrt{5}}y_{45}\left(O_{t}\right)+\frac{1}{5}y_{55}\left(O_{t}\right)+\frac{1}{\sqrt{35}}y_{57}\left(O_{t}\right)+\frac{1}{2\sqrt{10}}y_{58}\left(O_{t}\right)+\frac{1}{\sqrt{42}}y_{67}\left(O_{t}\right)\\ & \;+\frac{1}{7}y_{77}\left(O_{t}\right)+\frac{1}{2\sqrt{14}}y_{78}\left(O_{t}\right)+\frac{1}{8}y_{88}\left(O_{t}\right). \end{array}$$ (12)

Theorem 2.4. Let $O_{t}$ be a random cyclooctane chain of length *t* ≥ 3. Then

$$E^{F_{N}^{*}}\left(O_{t}\right)=1372+\left(t-2\right)\left[54p_{1}+4p_{2}\right]+\left(t-3\right)558.$$

Proof. For *t* = 3, we have $F_{N}^{*}\left(O_{3}^{p_{1}}\right)=1462$ , $F_{N}^{*}\left(O_{3}^{p_{2}}\right)=1376$ , $F_{N}^{*}\left(O_{3}^{p_{3}}\right)=1372$ , and $F_{N}^{*}\left(O_{3}^{p_{4}}\right)=1372$ . Hence, $E^{F_{N}^{*}}\left(O_{3}\right)=1372+54p_{1}+4p_{2}$ . For *t* ≥ 3, there are four possibilities

A) If $O_{t-1}\to O_{t}^{p_{1}}$ with probability *p*_1_, then $y_{44}\left(O_{t}^{p_{1}}\right)=y_{44}\left(O_{t-1}\right)+3$ , $y_{45}\left(O_{t}^{p_{1}}\right)=y_{45}\left(O_{t-1}\right)+2$ , $y_{58}\left(O_{t}^{p_{1}}\right)=y_{58}\left(O_{t-1}\right)+2$ and $y_{88}\left(O_{t}^{p_{1}}\right)=y_{88}\left(O_{t-1}\right)+2$ . The other *y*_*ij*_ values remains same. Using these values in 9, we have $F_{N}^{*}\left(O_{t}^{p_{1}}\right)=F_{N}^{*}\left(O_{t-1}^{p}\right)+612$ .
B) If $O_{t-1}\to O_{t}^{p_{2}}$ with probability *p*_2_, then $y_{44}\left(O_{t}^{p_{2}}\right)=y_{44}\left(O_{t-1}\right)+2$ , $y_{45}\left(O_{t}^{p_{2}}\right)=y_{45}\left(O_{t-1}\right)+2$ , $y_{57}\left(O_{t}^{p_{2}}\right)=y_{57}\left(O_{t-1}\right)+2$ , $y_{67}\left(O_{t}^{p_{2}}\right)=y_{67}\left(O_{t-1}\right)+2$ , and $y_{77}\left(O_{t}^{p_{2}}\right)=y_{77}\left(O_{t-1}\right)+1$ . The other *y*_*ij*_ values remains same. Using these values in 9, we have $F_{N}^{*}\left(O_{t}^{p_{2}}\right)=F_{N}^{*}\left(O_{t-1}\right)+562$ .
C) If $O_{t-1}\to O_{t}^{p_{3}}$ with probability *p*_3_, then $y_{44}\left(O_{t}^{p_{3}}\right)=y_{44}\left(O_{t-1}\right)+1$ , $y_{45}\left(O_{t}^{p_{3}}\right)=y_{45}\left(O_{t-1}\right)+2$ , $y_{55}\left(O_{t}^{p_{3}}\right)=y_{55}\left(O_{t-1}\right)+1$ , $y_{57}\left(O_{t}^{p_{3}}\right)=y_{57}\left(O_{t-1}\right)+4$ , and $y_{77}\left(O_{t}^{p_{3}}\right)=y_{77}\left(O_{t-1}\right)+1$ . The other *y*_*ij*_ values remains same. Using these values in 9, we have $F_{N}^{*}\left(O_{t}^{p_{3}}\right)=F_{N}^{*}\left(O_{t-1}\right)+558$ .
d) If $O_{t-1}\to O_{t}^{p_{4}}$ with probability *p*_4_, then $y_{44}\left(O_{t}^{p_{4}}\right)=y_{44}\left(O_{t-1}\right)$ , $y_{45}\left(O_{t}^{p_{4}}\right)=y_{45}\left(O_{t-1}\right)+4$ , $y_{57}\left(O_{t}^{p_{3}}\right)=y_{57}\left(O_{t-1}\right)+4$ , and $y_{77}\left(O_{t}^{p_{3}}\right)=y_{77}\left(O_{t-1}\right)+1$ . The other *y*_*ij*_ values remains same. Using these values in 9, we have $F_{N}^{*}\left(O_{t}^{p_{4}}\right)=F_{N}^{*}\left(O_{t-1}\right)+558$ .

Thus, we obtain

$$\begin{array}{ll} E^{F_{N}^{*}}\left(O_{t}\right) & =p_{1}F_{N}^{*}\left(O_{t}^{p_{1}}\right)+p_{2}F_{N}^{*}\left(O_{t}^{p_{2}}\right)+p_{3}F_{N}^{*}\left(O_{t}^{p_{3}}\right)+p_{4}F_{N}^{*}\left(O_{t}^{p_{4}}\right)\\ & =p_{1}\left(F_{N}^{*}\left(O_{t-1}\right)+612\right)+p_{2}\left(F_{N}^{*}\left(O_{t-1}\right)+562\right)+p_{3}\left(F_{N}^{*}\left(O_{t-1}\right)+558\right)\\ & \;+p_{4}\left(F_{N}^{*}\left(O_{t-1}\right)+558\right)\\ & =F_{N}^{*}\left(O_{t-1}\right)+558+54p_{1}+4p_{2}. \end{array}$$

By employing the operator *E* on both sides and considering the fact that $E\left[E^{F_{N}^{*}}\left(O_{t}\right)\right]=E^{F_{N}^{*}}\left(O_{t}\right)$ , we get

$$E^{F_{N}^{*}}\left(O_{t}\right)=E^{F_{N}^{*}}\left(O_{t-1}\right)+558+54p_{1}+4p_{2}.$$ (13)

Finally, solving the recurrence relation (13), we obtain

$$E^{F_{N}^{*}}\left(O_{t}\right)=1372+\left(t-2\right)\left[54p_{1}+4p_{2}\right]+558\left(t-3\right).$$

Theorem 2.5. Let $O_{t}$ be a random cyclooctane chain of length *t* ≥ 3. Then

EM2nmOt=26952240p1+1393411760p2+2296919600p3+45843920p4+t−359160p1+411811760p2+660919600p3+328980p4.

Proof. For *t* = 3, we have M2nm(O3p1)=26252240 , M2nm(O3p2)=1393411760 , M2nm(O3p3)=2296919600 and M2nm(O3p4)=45843920 . Hence, EM2nm(O3)=p126952240+p21393411760+p32296919600+p44584980 . For *t* > 3, there are four possibilities

A) If $O_{t-1}\to O_{t}^{p_{1}}$ with probability *p*_1_, then $y_{44}\left(O_{t}^{p_{1}}\right)=y_{44}\left(O_{t-1}\right)+3$ , $y_{45}\left(O_{t}^{p_{1}}\right)=y_{45}\left(O_{t-1}\right)+2$ and $y_{58}\left(O_{t}^{p_{1}}\right)=y_{58}\left(O_{t-1}\right)+2$ , and $y_{88}\left(O_{t}^{p_{1}}\right)=y_{88}\left(O_{t-1}\right)+2$ . The other *y*_*ij*_ values remains same. Using these values in 10, we have M2nm(Otp1)=M2nm(Ot−1)+59160 .
B) If $O_{t-1}\to O_{t}^{p_{2}}$ with probability *p*_2_, then $y_{44}\left(O_{t}^{p_{2}}\right)=y_{44}\left(O_{t-1}\right)+2$ , $x_{45}\left(O_{t}^{p_{2}}\right)=y_{45}\left(O_{t-1}\right)+2$ , $y_{57}\left(O_{t}^{p_{2}}\right)=y_{57}\left(O_{t-1}\right)+2$ , $y_{67}\left(O_{t}^{p_{2}}\right)=y_{67}\left(O_{t-1}\right)+2$ , and $y_{77}\left(O_{t}^{p_{2}}\right)=y_{77}\left(O_{t-1}\right)+1$ . The other *y*_*ij*_ values remains same. Using these values in 10, we have M2nm(Otp2)=M2nm(Ot−1p)+411811760 .
C) If $O_{t-1}\to O_{t}^{p_{3}}$ with probability *p*_3_, then $y_{44}\left(O_{t}^{p_{3}}\right)=y_{44}\left(O_{t-1}\right)+1$ , $y_{45}\left(O_{t}^{p_{3}}\right)=y_{45}\left(O_{t-1}\right)+2$ , $y_{55}\left(O_{t}^{p_{3}}\right)=y_{55}\left(O_{t-1}\right)+1$ , $y_{57}\left(O_{t}^{p_{3}}\right)=y_{57}\left(O_{t-1}\right)+4$ , and $y_{77}\left(O_{t}^{p_{3}}\right)=y_{77}\left(O_{t-1}\right)+1$ . The other *y*_*ij*_ values remains same. Using these values in 10, we have M2nm(Otp3)=M2nm(Ot−1p)+660919600 .
D) If $O_{t-1}\to O_{t}^{p_{4}}$ with probability *p*_4_, then $y_{44}\left(O_{t}^{p_{4}}\right)=y_{44}\left(O_{t-1}\right)$ , $y_{45}\left(O_{t}^{p_{4}}\right)=y_{45}\left(O_{t-1}\right)+4$ , $y_{57}\left(O_{t}^{p_{4}}\right)=y_{57}\left(O_{t-1}\right)+4$ and $y_{77}\left(O_{t}^{p_{4}}\right)=y_{77}\left(O_{t-1}\right)+1$ . The other *y*_*ij*_ values remains same. Using these values in 10, we have M2nm(Otp4)=M2nm(Ot−1p)+328980 .

Thus, we obtain

EM2nmOt=p1M2nmOtp1+p2M2nmOtp2+p3M2nmOtp3+p4M2nmOtp4=p1M2nmOt−1p+59160+p2M2nmOt−1p+411811760+p3M2nmOt−1p+660919600p4M2nmOt−1p+328980=M2nmOt−1p+p159160+p2411811760+p3660919600+p4328980.

By employing the operator *E* on both sides and considering the fact that EM2nm(Ot)=EM2nm(Ot) , we get

EM2nmOt=EM2nmOt−1+p1594480+p2411811760+p3660919600+p4328980. (14)

Finally, solving the recurrence relation (14), we obtain

EM2nmOt=26952240p1+1393411760p2+2296919600p3+45843920p4+t−359160p1+411811760p2+660919600p3+328980p4.

Theorem 2.6. Let $O_{t}$ be a random cyclooctane chain of length *t* ≥ 3. Then,

$$E^{ND_{3}}\left(O_{t}\right)=7196+716p_{1}+\left(t-3\right)\left[3086+746p_{1}\right]+\left(t-2\right)\left[148p_{2}+18p_{3}\right].$$

Proof. For *t* = 3, we have $ND_{3}\left(O_{3}^{p_{1}}\right)=7912$ , $ND_{3}\left(O_{3}^{p_{2}}\right)=7344$ , $ND_{3}\left(O_{3}^{p_{3}}\right)=7214$ and $ND_{3}\left(O_{3}^{p_{4}}\right)=7196$ . Hence, $E^{ND_{3}}\left(O_{3}\right)=7912p_{1}+7344p_{2}+7214p_{3}+7196p_{4}$ . For *t* > 3, there are four possibilities

A) If $O_{t-1}\to O_{t}^{p_{1}}$ with probability *p*_1_, then $y_{44}\left(O_{t}^{p_{1}}\right)=y_{44}\left(O_{t-1}\right)+3$ , $y_{45}\left(O_{t}^{p_{1}}\right)=y_{45}\left(O_{t-1}\right)+2$ , $y_{58}\left(O_{t}^{p_{1}}\right)=y_{58}\left(O_{t-1}\right)+2$ , and $y_{88}\left(O_{t}^{P_{1}}\right)=y_{88}\left(O_{t-1}\right)+2$ . The other *y*_*ij*_ values remains same. Using these values in 11, we have $ND_{3}\left(O_{t}^{p_{1}}\right)=ND_{3}\left(O_{t-1}\right)+3832$ .
B) If $O_{t-1}\to O_{t}^{p_{2}}$ with probability *p*_2_, then $y_{44}\left(O_{t}^{p_{2}}\right)=y_{44}\left(O_{t-1}\right)+2$ , $y_{45}\left(O_{t}^{p_{1}}\right)=y_{45}\left(O_{t-1}\right)+2$ , $y_{57}\left(O_{t}^{p_{2}}\right)=y_{57}\left(O_{t-1}\right)+2$ , $y_{67}\left(O_{t}^{p_{2}}\right)=y_{67}\left(O_{t-1}\right)+2$ , and $y_{77}\left(O_{t}^{p_{2}}\right)=y_{77}\left(O_{t-1}\right)+1$ . The other *y*_*ij*_ values remains same. Using these values in 11, we have $ND_{3}\left(O_{t}^{p_{2}}\right)=ND_{3}\left(O_{t-1}\right)+3234$ .
C) If $O_{t-1}\to O_{t}^{p_{3}}$ with probability *p*_3_, then $y_{44}\left(O_{t}^{p_{3}}\right)=y_{44}\left(O_{t-1}\right)+1$ , $y_{45}\left(O_{t}^{p_{3}}\right)=y_{45}\left(O_{t-1}\right)+2$ , $y_{55}\left(O_{t}^{p_{3}}=y_{55}\left(O_{t-1}\right)+1\right.$ , $y_{57}\left(O_{t}^{p_{3}}\right)=y_{57}\left(O_{t-1}\right)+4$ , and $y_{77}\left(O_{t}^{p_{3}}\right)=y_{77}\left(O_{t-1}\right)+1$ . The other *y*_*ij*_ values remains same. Using these values in 11, we have $ND_{3}\left(O_{t}^{p_{3}}\right)=ND_{3}\left(O_{t-1}^{p}\right)+3104$ .
D) If $O_{t-1}\to O_{t}^{p_{4}}$ with probability *p*_4_, then $y_{44}\left(O_{t}^{p_{4}}\right)=y_{44}\left(O_{t-1}\right)$ , $y_{45}\left(O_{t}^{p_{4}}\right)=y_{45}\left(O_{t-1}\right)+4$ , $y_{57}\left(O_{t}^{p_{3}}\right)=y_{57}\left(O_{t-1}\right)+4$ and $y_{77}\left(O_{t}^{p_{3}}\right)=y_{77}\left(O_{t-1}\right)+1$ . The other *y*_*ij*_ values remains same. Using these values in 11, we have $ND_{3}\left(O_{t}^{p_{4}}\right)=ND_{3}\left(O_{t-1}\right)+3086$ .

Thus, we obtain

$$\begin{array}{ll} E^{ND_{3}}\left(O_{t}\right) & =p_{1}ND_{3}\left(O_{t}^{p_{1}}\right)+p_{2}ND_{3}\left(O_{t}^{p_{2}}\right)+p_{3}ND_{3}\left(O_{t}^{p_{3}}\right)+p_{4}ND_{3}\left(O_{t}^{p_{4}}\right)\\ & =p_{1}\left(ND_{3}\left(O_{t-1}\right)+3832\right)+p_{2}\left(ND_{3}\left(O_{t-1}\right)+3234\right)+p_{3}\left(ND_{3}\left(O_{t-1}\right)+3104\right)\\ & \;+p_{4}\left(ND_{3}\right)\left(O_{t-1}\right)+3086\\ & =ND_{3}\left(O_{t-1}\right)+3832p_{1}+3234p_{2}+3104p_{3}+3086p_{4}. \end{array}$$

By employing the operator *E* on both sides and considering the fact that $E^{ND_{3}}\left(O_{t}\right)=E^{ND_{3}}\left(O_{t}\right)$ , we get

$$E^{ND_{3}}\left(O_{t}\right)=E^{ND_{3}}\left(O_{t-1}\right)+3234p_{1}+3234p_{2}+3104p_{3}+3086p_{4}.$$ (15)

Finally, solving the recurrence relation (15), we obtain

$$E^{ND_{3}}\left(O_{t}\right)=7196+716p_{1}+\left(t-3\right)\left[3086+746p_{1}\right]+\left(t-2\right)\left[148p_{2}+18p_{3}\right].$$

Theorem 2.7. Let $O_{t}$ be a random cyclooctane chain of length *t* ≥ 3. Then

$$\begin{array}{ll} E^{ND_{4}}\left(O_{t}\right) & =p_{1}\left[\frac{770+168\sqrt{5}+32\sqrt{35}+28\sqrt{10}+20\sqrt{14}+35}{280}\right]+p_{2}\left[\frac{585+126\sqrt{5}+36\sqrt{35}+10\sqrt{42}}{210}\right]\\ & \;+p_{3}\left[\frac{383+84\sqrt{5}+84\sqrt{35}}{140}\right]+p_{4}\left[\frac{80+28\sqrt{5}+8\sqrt{35}}{35}\right]\\ & \;+\left(t-3\right)\left[p_{1}\frac{10+2\sqrt{5}+\sqrt{10}}{10}+p_{2}\frac{177+12\sqrt{35}+10\sqrt{42}}{210}\right.\\ & \;+\left.p_{3}\frac{83+28\sqrt{5}+16\sqrt{35}}{140}+p_{4}\frac{5+14\sqrt{5}+4\sqrt{35}}{35}\right]. \end{array}$$

Proof. For *t* = 3, we have $ND_{4}\left(O_{3}^{p_{1}}\right)=\frac{770+168\sqrt{5}+32\sqrt{35}+28\sqrt{10}+20\sqrt{14}}{280}$ , $ND_{4}\left(O_{3}^{p_{2}}\right)=\frac{585+126\sqrt{5}+36\sqrt{35}+10\sqrt{42}}{210}$ , $ND_{4}\left(O_{3}^{p_{3}}\right)=\frac{383+84\sqrt{5}+84\sqrt{35}}{140}$ and $ND_{4}\left(O_{3}^{p_{4}}\right)=\frac{80+28\sqrt{5}+8\sqrt{35}}{35}$ . Hence,

$E^{ND_{4}}\left(O_{3}\right)=p_{1}\left[\frac{770+168\sqrt{5}+32\sqrt{35}+28\sqrt{10+20\sqrt{14}+35}}{280}\right]+p_{2}\left[\frac{585+126\sqrt{5}+36\sqrt{35}+10\sqrt{42}}{210}\right]+p_{3}\left[\frac{383+84\sqrt{5}+84\sqrt{35}}{140}+p_{4}\frac{5+14\sqrt{5}+4\sqrt{35}}{35}\right]$ . For *t* > 3 there are four possibilities

A) If $O_{t-1}\to O_{t}^{p_{1}}$ with probability *p*_1_, then $y_{44}\left(O_{t}^{p_{1}}\right)=y_{44}\left(O_{t-1}^{p}\right)+3$ , $y_{45}\left(O_{t}^{p_{1}}\right)=y_{45}\left(O_{t-1}\right)+2$ , $y_{58}\left(O_{t}^{p_{1}}\right)=y_{58}\left(O_{t-1}\right)+2$ , and $y_{88}\left(O_{t}^{p_{1}}\right)=y_{88}\left(O_{t-1}\right)+2$ . The other *y*_*ij*_ values remains same. Using these values in 12, we have $ND_{4}\left(O_{t}^{p_{1}}\right)=ND_{4}\left(O_{t-1}\right)+\frac{10+2\sqrt{5}+\sqrt{10}}{10}$ .
B) If $O_{t-1}\to O_{t}^{p_{2}}$ with probability *p*_2_, then $y_{44}\left(O_{t}^{p_{2}}\right)=y_{44}\left(O_{t-1}\right)+2$ , $y_{45}\left(O_{t}^{p_{1}}\right)=y_{45}\left(O_{t-1}\right)+2$ , $y_{57}\left(O_{t}^{p_{2}}\right)=y_{57}\left(O_{t-1}\right)+2$ , $y_{67}\left(O_{t}^{p_{2}}\right)=y_{67}\left(O_{t-1}\right)+2$ , and $y_{77}\left(O_{t}^{p_{2}}\right)=y_{77}\left(O_{t-1}\right)+1$ . The other *y*_*ij*_ values remains same. Using these values in 12, we have $ND_{4}\left(O_{t}^{p_{2}}\right)=ND_{4}\left(O_{t-1}^{p}\right)+\frac{177+12\sqrt{35}+10\sqrt{42}}{210}$ .
C) If $O_{t-1}\to O_{t}^{p_{3}}$ with probability *p*_3_, then $y_{44}\left(O_{t}^{p_{3}}\right)=y_{44}\left(O_{t-1}\right)+1$ , $y_{45}\left(O_{t}^{p_{3}}\right)=y_{45}\left(O_{t-1}\right)+2$ , $y_{55}\left(O_{t}^{p_{3}}\right)=y_{55}\left(O_{t-1}\right)+1$ , $y_{57}\left(O_{t}^{p_{3}}\right)=y_{57}\left(O_{t-1}\right)+4$ , and $y_{77}\left(O_{t}^{p_{3}}\right)=y_{77}\left(O_{t-1}\right)+1$ . The other *y*_*ij*_ values remains same. Using these values in 12, we have $ND_{4}\left(O_{t}^{p_{3}}\right)=ND_{4}\left(O_{t-1}\right)+\frac{83+28\sqrt{5}+16\sqrt{35}}{140}$ .
D) If $O_{t-1}\to O_{t}^{p_{4}}$ with probability *p*_4_, then $y_{44}\left(O_{t}^{p_{4}}\right)=y_{44}\left(O_{t-1}\right)$ , $y_{45}\left(O_{t}^{p_{4}}\right)=y_{45}\left(O_{t-1}\right)+4$ , $y_{57}\left(O_{t}^{p_{3}}\right)=y_{57}\left(O_{t-1}\right)+4$ , and $y_{77}\left(O_{t}^{p_{3}}\right)=y_{77}\left(O_{t-1}\right)+1$ . The other *y*_*ij*_ values remains same. Using these values in 12, we have $ND_{4}\left(O_{t}^{p_{4}}\right)=ND_{4}\left(O_{t-1}\right)+\frac{5+14\sqrt{5}+4\sqrt{35}}{35}$ .

Thus, we obtain

$$\begin{array}{ll} E^{ND_{4}}\left(O_{t}\right) & =p_{1}ND_{4}\left(O_{t}^{p_{1}}\right)+p_{2}ND_{4}\left(O_{t}^{p_{2}}\right)+p_{3}ND_{4}\left(O_{t}^{p_{3}}\right)+p_{4}ND_{4}\left(O_{t}^{p_{4}}\right)\\ & =p_{1}ND_{4}\left(O_{t-1}\right)+\frac{10+2\sqrt{5}+\sqrt{10}}{10}+p_{2}ND_{4}\left(O_{t-1}\right)+\frac{177+12\sqrt{35}+10\sqrt{42}}{210}\\ & \;+p_{3}ND_{4}\left(O_{t-1}\right)+\frac{83+28\sqrt{5}+16\sqrt{35}}{140}+p_{4}ND_{4}\left(O_{t-1}\right)+\frac{5+14\sqrt{5}+4\sqrt{35}}{35}\\ & =ND_{4}\left(O_{t-1}\right)+p_{1}\left[\frac{10+2\sqrt{5}+\sqrt{10}}{10}\right]+p_{2}\left[\frac{177+12\sqrt{35}+10\sqrt{42}}{210}\right]\\ & \;+p_{3}\left[\frac{83+28\sqrt{5}+16\sqrt{35}}{140}\right]+p_{4}\left[\frac{5+14\sqrt{5}+4\sqrt{35}}{35}\right]. \end{array}$$

By employing the operator *E* on both sides and considering the fact that $E^{ND_{4}}\left(O_{t}\right)=E^{ND_{4}}\left(O_{t}\right)$ , we get

$$\begin{array}{ll} E^{ND_{4}}\left(O_{t}\right) & =E^{ND_{4}}\left(O_{t-1}\right)+p_{1}\left[\frac{10+2\sqrt{5}+\sqrt{10}}{10}\right]+p_{2}\left[\frac{177+12\sqrt{35}+10\sqrt{42}}{210}\right]\\ & \;+p_{3}\left[\frac{83+28\sqrt{5}+16\sqrt{35}}{140}\right]+p_{4}\left[\frac{5+14\sqrt{5}+4\sqrt{35}}{35}\right]. \end{array}$$ (16)

Finally, solving the recurrence relation (16), we obtain

$$\begin{array}{ll} E^{ND_{4}}\left(O_{t}\right) & =p_{1}\frac{770+168\sqrt{5}+32\sqrt{35}+28\sqrt{10}+20\sqrt{14}+35}{280}\\ & \;+p_{2}\frac{585+126\sqrt{5}+36\sqrt{35}+10\sqrt{42}}{210}\\ & \;+p_{3}\frac{383+84\sqrt{5}+84\sqrt{35}}{140}+p_{4}\frac{80+28\sqrt{5}+8\sqrt{35}}{35}\\ & \;+\left(t-3\right)\left[p_{1}\frac{10+2\sqrt{5}+\sqrt{10}}{10}+p_{2}\frac{177+12\sqrt{35}+10\sqrt{42}}{210}\right.\\ & \;+\left.p_{3}\frac{83+28\sqrt{5}+16\sqrt{35}}{140}+p_{4}\frac{5+14\sqrt{5}+4\sqrt{35}}{35}\right]. \end{array}$$

Corollary 2.8. *For* *t* ≥ 3*, we have*

1. • $EF_{N}^{*}\left(CO_{t}^{p}\right)=612t-310$ .
  • $\;EF_{N}^{*}\left(ZO_{t}^{p}\right)=562t-310$ .
  • $\;EF_{N}^{*}\left(MO_{t}^{p}\right)=588t-302$ .
  • $\;EF_{N}^{*}\left(LO_{t}^{p}\right)=588t-302$ .
2. • $E^{nm}M_{2}\left(CO_{t}^{p}\right)=\frac{59}{160}t+\frac{217}{2240}$ .
  • ${\;E}^{nm}M_{2}\left(ZO_{t}^{p}\right)=\frac{4118}{11760}t-\frac{79}{588}$ .
  • ${\;E}^{nm}M_{2}\left(MO_{t}^{p}\right)=\frac{6609}{19600}t+\frac{1571}{9800}$ .
  • ${\;E}^{nm}M_{2}\left(LO_{t}^{p}\right)=\frac{328}{90}t-\frac{81}{490}$ .
3. • $END_{3}\left(CO_{t}^{p}\right)=3832t-3584$ .
  • $\;END_{3}\left(ZO_{t}^{p}\right)=3234t-2358$ .
  • $\;END_{3}\left(MO_{t}^{p}\right)=3104t-2098$ .
  • $\;END_{3}\left(LO_{t}^{p}\right)=3086t-2062$ .
4. • $\;END_{4}\left(CO_{t}^{p}\right)=\left[\frac{770+168\sqrt{5}+32\sqrt{35}+28\sqrt{10}+20\sqrt{14}+35}{280}\right]+\left(t-3\right)\left[\frac{10+2\sqrt{5}+\sqrt{10}}{10}\right]$ .
  • $\;END_{4}\left(ZO_{t}^{p}\right)=\left[\frac{128+126\sqrt{5}+36\sqrt{35}+10\sqrt{42}}{210}\right]+\left(t-3\right)\left[\frac{177+12\sqrt{35}+10\sqrt{42}}{210}\right]$ .
  • $\;END_{4}\left(MO_{t}^{p}\right)=\left[\frac{383+84\sqrt{5}+32\sqrt{35}}{140}\right]+\left(t-3\right)\left[\frac{83+28\sqrt{35}+16\sqrt{35}}{140}\right]$ .
  • $\;END_{4}\left(LO_{t}^{p}\right)=\left[\frac{84+28\sqrt{5}+8\sqrt{35}}{35}\right]+\left(t-3\right)\left[\frac{5+14\sqrt{5}+4\sqrt{35}}{35}\right]$ .

## 3 A comparison between the expected values of topological indices for random cyclooctane chains

**FIGURE 5:**
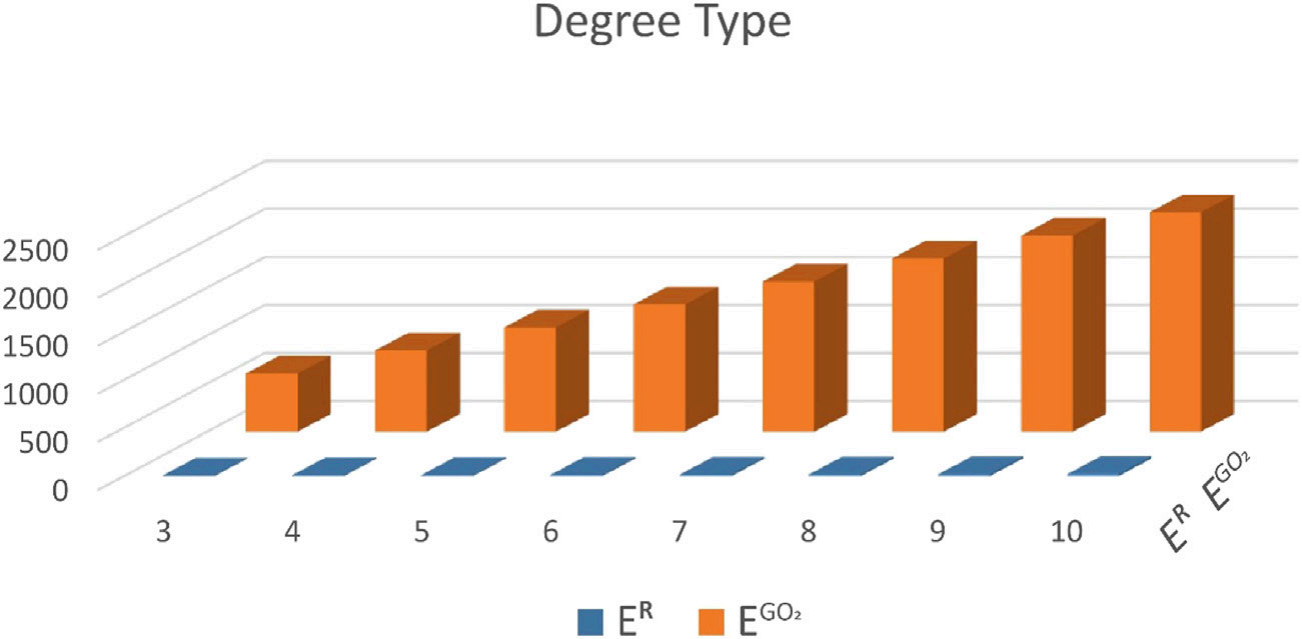
Graphical comparison of *E* ^*R*^ and $E^{GO_{2}}$ .

In this section, we give a comparison between the expected values of the considered topological indices for Random Cyclooctane Chains. In Table 1, we have calculated the expected values of the forgotten index and the second Gourava index of random cyclooctane chains for *t* = 3, 4, … , 10. The plot of the expected values of these topological indices for different values of *t* is depicted in Figure 5. Observe that the expected values for the forgotten index are always less than the expected value of the second Gourava index for all *t* ≥ 3. Now, we give an explicit proof of the fact that for any *t* ≥ 3 the expected value of second Gourava index is always greater than the expected value of the forgotten index in random cyclooctane chains.

**TABLE 1:** Expected values *E* ^*R*^ and $E^{GO_{2}}$ .

*t*	*E* ^*R*^	$E^{GO_{2}}$
3	12.2693	606
4	16.5709	846
5	20.8725	1086
6	25.1741	1326
7	29.4757	1566
8	33.7773	1806
9	38.0789	2046
10	42.3805	2286

Theorem 3.1. *Let* *t* ≥ 3*, then* $\left[E^{GO_{2}}\left(O_{t}\right)\right]>\left[E^{R}\left(O_{t}\right.\right]$

Proof. For *t* = 3, the result follows immediately. By applying Theorems 2.1 and 2.2, we get

$$\begin{array}{ll} \left[E^{GO_{2}}\left(O_{t}\right)\right]-\left[E^{R}\left(O_{t}\right.\right] & =366+\left(t-2\right)\left(238+10p_{1}\right)-\frac{19+2\sqrt{6}}{3}+\left(t-2\right)\left[\frac{7+2\sqrt{6}}{3}\right.\\ & \;+\left.\left(\frac{15-2\sqrt{6}}{6}\right)p_{1}\right]\\ & =\left(t-2\right)\left[\left(238+10p_{1}\right)-\left(\frac{7+2\sqrt{6}}{3}+\left(\frac{15-2\sqrt{6}}{6}\right)p_{1}\right)\right]\\ & \;+366-\left(\frac{19+2\sqrt{6}}{3}\right)\\ & =\left(t-2\right)\left[238-\left(\frac{7+2\sqrt{6}}{3}\right)+\left(10-\left(\frac{15-2\sqrt{6}}{6}\right)\right.p_{1}\right]\\ & \;+366-\left(\frac{19+2\sqrt{6}}{3}\right)>0\;\;\;\;\;\;\;\;\;∵t>2. \end{array}$$

**FIGURE 6:**
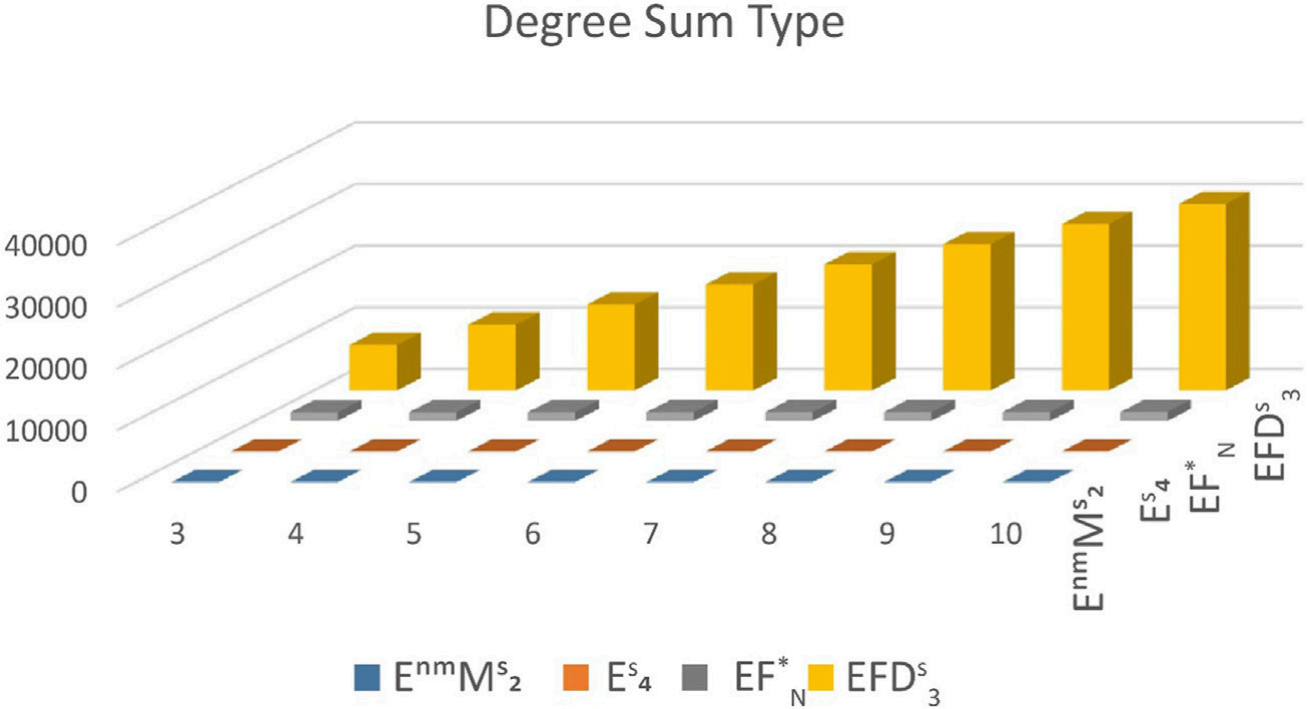
Graphical comparison of EM2nm , $E^{ND_{4}}$ , $E^{F_{N}^{*}}$ and $E^{ND_{3}}$ .

Next, we give a comparison between the expected values of the topological indices based on the neighborhood degree for cyclooctane chains. The expected values of the modified neighborhood Forgotten index, the modified neighborhood second Zagreb index, the third neighborhood index, and the fourth neighborhood index are calculated and depicted in Table 2 for *t* = 3, 4, … , 10. A 2D plot of these values is shown in Figure 6. Observe that EM2nm<END4<EFN*<END3 . Now, we give explicit proof of this fact.

**TABLE 2:** Expected values of EM2nm , $E^{ND_{4}}$ , $E^{F_{N}^{*}}$ and $E^{ND_{3}}$ .

*t*	EM2nm	$E^{ND_{4}}$	$E^{F_{N}^{*}}$	$E^{ND_{3}}$
3	1.17974	5.4420	1383.6	7372.4
4	1.5249	7.4638	1395.2	10640.8
5	1.861	9.4856	1406.8	13909.2
6	2.2151	11.5074	1418.4	17177.6
7	2.5602	13.5292	1430	20446
8	2.9053	15.5509	1431.6	23714.4
9	3.2504	17.5728	1443.2	26982.8
10	3.5955	19.5946	1454.8	30251.2

Theorem 3.2. *Let* *t* ≥ 3*, then* [EM2nm]<[END4]

Proof. For *t* = 3, the result follows immediately. By applying Theorems 2.7 and 2.5, we get

END4−EM2nm=t−31.7634−0.3688p1+1.4895−0.3502p2+1.7162−0.3372p3+1.7134−0.3347p4+5.4763−1.2031p1+5.4501−1.18486p2+7.6270−1.17188p3+5.4269−1.1694p4=t−31.3946p1+1.1393p2+1.379p3+1.3787p4+4.2732p1+4.2652p2+6.4551p3+4.2875p4>0∵t>3.

Theorem 3.3. *Let* *t* ≥ 3*, then* $E^{ND_{4}}<E^{F_{N}^{*}}$

Proof. For *t* = 3, the result follows immediately. By applying Theorems 2.7 and 2.4, we get

$$\begin{array}{ll} \left[E^{F_{N}^{*}}\right]-\left[E^{ND_{4}}\right] & =\left(t-3\right)\left[558-1.7634p_{1}-1.4895p_{2}-1.7162p_{3}\right.\\ & \left.\;-1.7134p_{4}\right]+\left(t-2\right)\left[54p_{1}+4p_{2}\right]+1372>0\;\;\;\;\;\;\;\;\;∵t>3. \end{array}$$

Theorem 3.4. *For* *t* ≥ 3*, we have*

$$\left[E^{F_{N}^{*}}\right]<\left[E^{ND_{3}}\right].$$

Proof. For *t* = 3, the result follows immediately. So, we get

$$\begin{array}{ll} \left[E^{ND_{3}}\right]-\left[E^{F_{N}^{*}}\right] & =\left(t-3\right)\left[3086+746p_{1}-558\right]+\left(t-2\right)\left[144p_{2}+18p_{3}-54p_{1}\right]\\ & \;+7196+716p_{2}-1372>0\;\;\;\;\;\;\;\;\;∵t>3. \end{array}$$

## 4 Conclusion

In this paper, we have studied the behaviour of cyclooctane chains and calculated the expected values of the neighbourhood sum of some topological indices, which are the neighbourhood forgotten index, general Randić index, modified second Zagreb index, and third, fourth and fifth (NDe) indices of cyclooctane chains. It has been observed that the neighbourhood third (NDe) index has highest value and neighbourhood modified second Zagreb descriptor has the smallest value. In addition, the expected values of cyclooctane chains in some special cases have been computed. The results may be helpful in studying various physical/chemical properties of cyclooctane chains.

## Data Availability

The original contributions presented in the study are included in the article/Supplementary material, further inquiries can be directed to the corresponding author.
